# Avian Sex Determination: A Chicken and Egg Conundrum

**DOI:** 10.1159/000529754

**Published:** 2023-02-16

**Authors:** Michael Clinton, Debiao Zhao

**Affiliations:** Roslin Institute Chicken Embryology (RICE) Group, Gene Function and Development, The Roslin Institute, University of Edinburgh, Easter Bush Campus, Midlothian, UK

**Keywords:** Chicken, Gonads, DMRT1, Estrogen

## Abstract

**Background:**

Primary sex determination is the developmental process that results in the sexual differentiation of the gonads. Vertebrate sex determination is generally considered to follow the model based on the mammalian system, where a sex-specific master regulatory gene activates one of the two different gene networks that underlie testis and ovary differentiation.

**Summary:**

It is now known that, while many of the molecular components of these pathways are conserved across different vertebrates, a wide variety of different trigger factors are utilized to initiate primary sex determination. In birds, the male is the homogametic sex (ZZ), and significant differences exist between the avian system of sex determination and that of mammals. For example, DMRT1, FOXL2, and estrogen are key factors in gonadogenesis in birds, but none are essential for primary sex determination in mammals.

**Key Message:**

Gonadal sex determination in birds is thought to depend on a dosage-based mechanism involving expression of the Z-linked DMRT1 gene, and it may be that this “mechanism” is simply an extension of the cell autonomous sex identity associated with avian tissues, with no sex-specific trigger required.

## Introduction

Primary sex determination initiates gonadal sex differentiation, a process whereby the undifferentiated gonad develops as either a testis or an ovary. This is thought to require expression of a master regulator gene that triggers expression of a gene cascade in the bipotential gonad precursor in one sex. The selection of a particular developmental pathway is also considered to suppress the alternative developmental pathway (often considered the default option). This review will consider how well these principles apply to gonad sex determination and differentiation in the chicken.

## Morphological Development

**Fig. 1.**
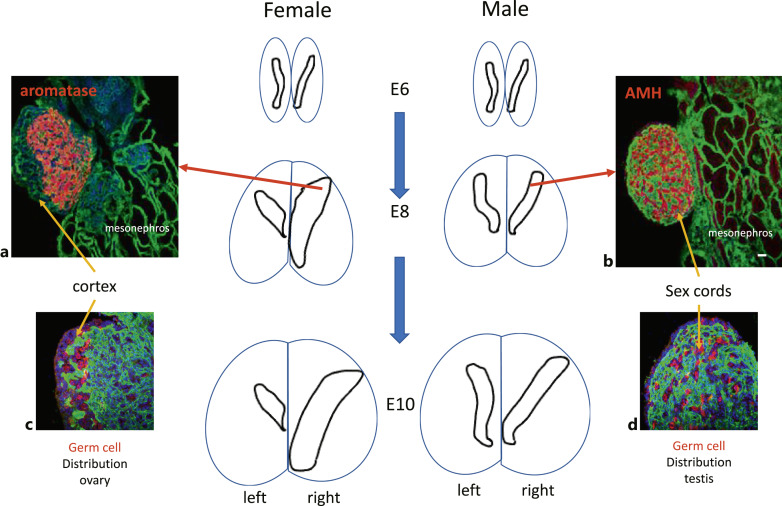
Schematic illustrating morphological development of female and male gonads. Immunohistochemistry (IHC) on histological sections through gonads and mesonephros illustrates aromatase expression (red) throughout the female medulla (**a**) and AMH expression (red) in the developing sex cords of the male medulla (**b**). IHC with VASA antibody (red) shows that by E10, germ cells are located in the cortex of the female gonad (**c**) and within the sex cords of the male gonad (**d**).

In the chicken, the gonads initially arise around embryonic day 3 [Ayers et al., 2013c] of development (equivalent to H&H developmental stage 23 [Hamburger and Hamilton, 1951]) and are macroscopically evident around E4.5. The gonads appear as a ridge of thickened coelomic epithelium running in an anterior posterior orientation on the ventral surface of each mesonephros. In the initial stages of gonadogenesis (E4.5–E6.5), these genital ridges appear identical in male and female embryos. Histologically, the gonads are composed of two elements, an inner core containing cellular condensations known as primitive/primary sex cords surrounded by a thin outer layer of columnar epithelium cells. As with similar bipartite organs, the inner core is designated the medulla and the outer layer, the cortex. By E8, the gonads in male and female embryos are distinctly different (Fig. 1). In males, the right and left gonads adopt a tubular structure, and while the right female gonad is similar in appearance but smaller than the male gonads, the left female gonad is broader and flatter and considerably larger [Guioli et al., 2014]. In males, both right and left testes comprise a thin simple epithelium surrounding a core medulla organized into branched tubular structures known as sex cords (Fig. 1). These sex cords are composed of differentiating Sertoli cells and germ cells, encased within a basement membrane. The differentiating Sertoli cells express anti-Mullerian hormone (AMH) and later SRY-box 9 (SOX9) [Oreal et al., 1998, 2002; Majorand Smith et al., 2016]. By this stage in females, the outer epithelial layers of the right and left gonads are markedly different: while the right ovary is surrounded by a thin epithelial layer, the left ovary is encased in a thick sheath of cells, designating the cortex. The medulla of both ovaries is similar, appears largely unstructured, and contains fluid-filled vacuoles known as lacunae (possibly degenerating primitive sex cords). FOXL2 and aromatase (CYP19A1) expression is widespread throughout the medulla of both ovaries [Smith et al., 1997; Shimada, 1998]. In female chickens (and most birds), only the left gonad develops into a fully functional ovary, while the right gonad – although steroidogenically active during embryogenesis – regresses post-hatch.

Avian germ cells are most likely specified by preformation from primordial germ cell (PGC) precursors during the earliest stages of embryo development [Tsunekawa et al., 2000; Lee et al., 2016]. At HH stage 4, PGCs move into an extra-embryonic region known as the germinal crescent and at HH stages 13–17 are transported to the gonadal region via the circulation. By E6.5, germ cells are randomly distributed throughout both male gonads and the right female gonad, but in the left female gonad the germ cells are located close to the epithelium [Guioli et al., 2014]. By E10.5, the majority of germ cells in the left female gonad are encased within an expanded cortex (Fig. 1). Prior to hatch, in the left ovary large numbers of germ cells are located almost exclusively in the cortex, while in the right ovary a smaller number is randomly distributed throughout the medulla. At this stage in the male, similar numbers of germ cells are located in the medullary sex cords of both testes.

## Sex Chromosomes

Birds differ from mammals in that the female is the heterogametic sex, with one Z sex chromosome and one W sex chromosome, while males are the homogametic sex with two Z chromosomes. The designations Z and W have no particular significance other than to distinguish them from the mammalian X and Y, but of course under this system the female parent’s contribution determines the sex of the offspring. As in mammals, the heterogametic chromosomes comprise a major chromosome (Z) and a smaller, largely degraded, partner (W) chromosome [Marshall Graves and Shetty, 2001]. The Z chromosome encodes approximately 1,000 protein coding genes, and the W chromosome encodes around 30 protein coding homologues and a small number of heavily repeated sequences [Ayers et al., 2013b] [Genome Reference Consortium Chicken Build 6a].

## Dosage Compensation in Chickens

Male chicken cells lack sex chromatin, and there is no evidence of a late-replicating Z chromosome, indicating that the mammalian form of sex chromosome inactivation is unlikely. Indeed, until relatively recently, it was widely believed that the chicken sex chromosomes were not subject to any form of dosage compensation. This was largely based on evidence that the levels of activity of the Z-linked liver enzyme, aconitase (IREBP), were higher in males than in females [Baverstock et al., 1982]. The first evidence in favor of an avian dosage compensation system came from a study that examined transcription of a small number of Z-linked genes in male and female embryos [McQueen et al., 2001]. This study examined expression prior to the point of sex determination to exclude any possible influence of gonadal hormones. This analysis established that, for six of the nine genes studied, expression levels were equivalent in male and female embryos, while expression of the remaining three genes was higher in males than in females. This showed that the expression of chicken sex chromosome genes was subject to some form of equalization, and that this did not apply to all Z chromosome genes. Since then, a large number of high-throughput studies that examined the expression of the majority of Z-linked genes have been carried out, confirming and extending these findings [Ayers et al., 2013a; Ellegren et al., 2007; Melamed and Arnold, 2007; Arnold et al., 2008]. It is clear that equalization of Z-linked gene expression is not chromosome-wide in birds, and it has been suggested that this “dosage compensation” is applied on a gene-by-gene basis [Mank and Ellegren, 2009]. It may be that this “variable compensation” underlies the strong cell autonomous sex identity (CASI) associated with chicken somatic tissues [Zhao et al., 2010].

## Sex-Determining Mechanism

The first sex-determining gene to be identified among the vertebrates was the mammalian Sry gene (sex-determining region on the Y chromosome) (reviewed in Kashimada and Koopman, 2010; Sekido, 2010). This discovery spurred the search for Sry homologues in other vertebrates, and although a number of related SRY-box (SOX) genes were found to be expressed in chicken gonads [McBride et al., 1997], Sry proved to be mammalian-specific. It is now established that vertebrates display a range of sex-determining mechanisms, and a diverse variety of sex-determining genes have been identified. These include amhy, Amhr2, DMY, dmrt1by, dmrt1 (fish and reptiles), DM-W, and Z-AR (amphibians) [Hattori et al., 2012; Kamiya et al., 2012; Matsuda et al., 2002; Nanda et al., 2002; Cui et al., 2017; Yoshimoto et al., 2008; Oike et al., 2017]. Although the downstream components of the gonadal differentiation pathways are generally well conserved among vertebrates, the master regulator that “switches on” the pathways is highly divergent. In the case of the chicken, two versions of genetic sex determination have regularly been proposed as potential avian sex-determining mechanisms [Clinton, 1998; Smith and Sinclair, 2004; Kuroiwa, 2017]. The first postulates the presence of an ovary-determining gene located on the female-specific W chromosome akin to the mammalian testis-determining SRY gene. However, attempts to identify a suitable candidate ovary-determining gene have proved unsuccessful [Hirst et al., 2017; Lui et al., 2019], and recent evidence suggests that W chromosome genes are not required for ovary development [Ioannidis et al., 2021]. The second proposed mechanism is that primary sex determination depends on a gene dosage mechanism based on a Z chromosome gene, and for some time, evidence has accumulated that the DMRT1 gene is the most likely candidate for such a mechanism [Nanda et al., 1999; Smith et al., 1999]. At the time of gonadal sex determination, DMRT1 is expressed at higher levels in the male embryo than the female embryo and expression is restricted to the gonads and the Mullerian ducts [Raymond et al., 1999; Smith et al., 2003; Omotehara et al., 2014]. Manipulating DMRT1 levels in the developing chick gonad by siRNA and RCAS virus approaches has shown that over-expression of DMRT1 leads to masculinization of the genetically female (ZW) gonad, and that reducing DMRT1 levels results in feminization of the genetically male (ZZ) gonads [Smith et al., 2009; Lambeth et al., 2014; Cooper et al., 2017]. More recently, a CRISPR-cas9 approach was used to disable a single copy of DMRT1 in ZZ PGCs [Ioannidis et al., 2021]. These targeted PGCs were injected into surrogate host chicken embryos from which the endogenous germ cells had been ablated. The surrogate host chicks were hatched and raised to sexual maturity and mated to wild-type female birds, producing male birds that were heterozygous for functional DMRT1 and female birds that lacked functional DMRT1. The genetically male (ZZ) birds with only a single functional copy of DMRT1 developed ovaries typical of those of a female bird. These gonads expressed high levels of typical female marker genes (FOXL2 and aromatase) and lacked expression of typical male marker genes (AMH and SOX9). This analysis confirmed that DMRT1 was indeed a key factor in avian gonadal sex determination and suggested that two functional copies were required for testis development in males. The loss of one copy of DMRT1 in genetically male embryos clearly leads to the induction of the gene network underlying ovary development. The temporal and spatial expression of FOXL2 and then aromatase is identical to that seen in the gonads of wild-type female embryos. This study suggested that in wild-type male embryos, the presence of two functional copies of DMRT1, either directly or indirectly, suppresses expression of FOXL2. So in wild-type male embryos, DMRT1 stimulates the testis development pathway and inhibits the ovary development pathway. The fact that genetically male birds can form a typical ovary also indicates that W chromosome genes are not required for ovary development (although this does not exclude the possibility that W chromosome genes may be involved in suppressing testis development in wild-type female birds). Interestingly, genetically female birds that lacked functional DMRT1 also developed ovaries, suggesting that DMRT1 is not required for initiating the ovarian pathway. However, these ovaries were obviously smaller than typical ovaries, perhaps indicating that DMRT1 is required for growth of the gonadal medulla [Ioannidis et al., 2021].

**Fig. 2.**
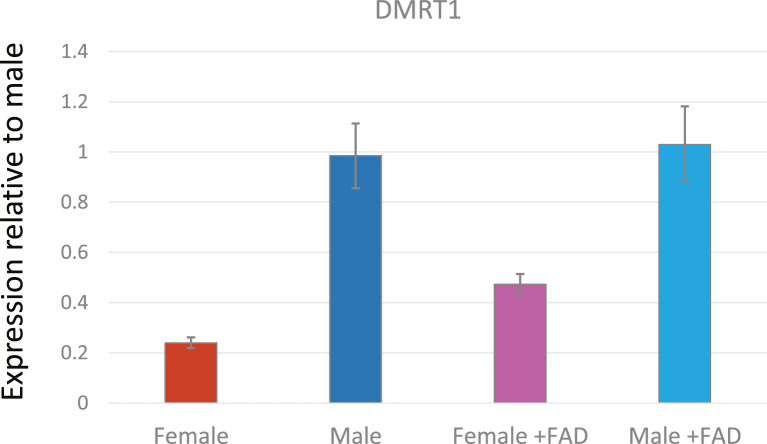
Relative expression of DMRT1 in gonads of wild-type and fadrozole-treated chick embryos. Q-PCR quantitation of DMRT1 transcripts in gonads of E7.5 female and male embryos, and in fadrozole-treated (FAD) female and male embryos. Embryos were injected with either PBS or fadrozole at E2.5 and collected for gonad dissection at E7.5. A minimum of five gonad pairs were pooled for each sample type, and five individual pools were analyzed for each sample type. Expression levels are shown relative to a single male pool. Bars represent the mean value ± 1 SD.

**Fig. 3.**
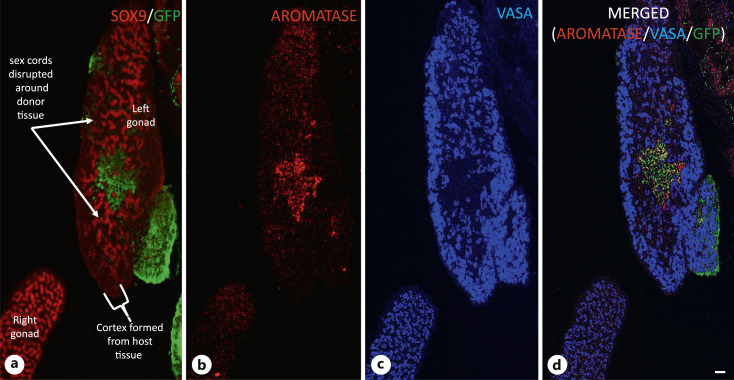
Expression of male and female “marker” genes in mixed-sex chimeric gonads. IHC showing expression of SOX9 (**a**/red), aromatase (**b**/red), and VASA (**c**/blue) in adjacent longitudinal sections of right and left gonads of a mixed-sex chimera. Chimera was generated by transplantation of lateral plate mesoderm (LPM) from left side of female donor into left side of male host. GFP indicates contribution of female donor. The left male gonad is surrounded by an obvious cortical layer that is absent in the right gonad. Only a small portion of the cortex is derived from the female donor (shown in green), while the majority is derived from male host tissue. The majority of germ cells (blue) in the left gonad are located in the cortex as is typical of an ovary, while the majority of the germ cells in the right gonad are located in the SOX9 +ve sex cords. Sex cord development in the left male gonad is clearly disrupted in the region around the aromatase-expressing donor tissue. **d** Panels **a–c** merged.

It is tempting to consider the role of a double dose of DMRT1 in chick sex determination as equivalent to the role of SRY in the mammalian model: i.e., a master regulator that initiates testis development and leads to the induction of AMH and SOX9, and that without this double dose, gonadal development follows a default ovarian pathway. However, evidence that estrogens play a major role in gonadal development in the chicken suggests that the process is not so straightforward. Indeed, if estrogen synthesis is blocked in the male embryos heterozygous for functional DMRT1, testicular development is “rescued.” This suggests that estrogens inhibit the expression/action of DMRT1, and confirms that a single functional copy of DMRT1 is sufficient for testes development, as previously proposed by Smith et al. [2003]. Numerous studies have highlighted the importance of estrogen in avian gonadal sex determination [Scheib, 1983; Elbrecht and Smith, 1992; Smith et al., 2003; Hudson et al., 2005; Guioli et al., 2020]: estrogen treatment of wild-type male embryos leads to ovary development, and inhibition of estrogen synthesis in wild-type female embryos leads to ovary-to-testis sex-reversal and increased expression of DMRT1. While the expression of DMRT1 from the single copy is increased in sex-reversed female embryos, the expression levels are not equivalent to those seen in wild-type males (Fig. 2). The effect of estrogens is also demonstrated by a study of embryos with mixed-sex chimeric gonads, i.e., gonads composed of a mixture of male (ZZ) and female (ZW) tissues. In this approach, lateral plate mesoderm (LPM) is removed from an embryo of one sex and transplanted into an embryo of the opposite sex [Zhao et al., 2010]. This procedure is carried out in ovo at embryonic day 2, and the egg is resealed and re-incubated and then examined at a later stage of development. Cells from the transplanted donor tissue proliferate, migrate, and differentiate in an identical manner to the cells of the endogenous LPM and eventually form part of the host gonad. A series of transplantations were carried out to generate embryos with mixed-sex chimeric gonads, and these were analyzed at E9 and E19. A minimum of two examples of all sixteen possible male/female and left/right combinations were generated for both embryonic stages. A summary of the effects of transplantation on cortex formation in donor and host tissue is detailed in online supplementary Table 1 (for all online suppl. material, see www.karger.com/doi/10.1159/000529754). This analysis revealed that a relatively small portion of aromatase-expressing female donor tissue present in the left gonad of a male host was capable of inducing cortex formation in the male. Most convincing, cells that are normally destined to form the female right gonad (and normally incapable of forming an ovarian cortex), when transplanted into male embryos, induced ovary development of the male left gonad. In the example illustrated (Fig. 3), the small quantity of female donor tissue present in the medulla of the male host not only induces a “male cortex,” but also disrupts the formation of sex cords in the surrounding male tissue. This suggests that in female gonads, estrogens are not only responsible for inducing cortex formation, but also inhibit sex cords/testis development in the medulla. Of course, the key to the influence of estrogen on gonadal development lies in the distribution of the estrogen receptor (ERα) [Smith et al., 1997; Guioli and Lovell-Badge, 2007; Zhao et al., 2022]. ERα protein is expressed in the medulla of both right and left gonads, but only in the outer epithelium of the left gonad. Most importantly, ERα is expressed in the epithelium of the left gonad of both male and female embryos. In the presence of estrogen, ER binds the steroid, translocates to the nucleus, and induces expression of downstream target genes. In mixed-sex chimeras, estrogen synthesized by the female portion activates ERα in the epithelium of the male portion and induces cortex formation [Guioli et al., 2020]. It is clear that in the male left gonad, estrogen is able to over-ride the testis-promoting influence of a double dose of DMRT1.

So it seems that, in male embryos, DMRT1 is essential for initiating the testis development pathway and appears to inhibit the ovary development pathway. Conversely, in female embryos, estrogen is required to maintain the ovarian pathway and may be capable of suppressing the testis pathway.

Estrogen has also been shown to be an important factor in gonadogenesis in other vertebrates. In the African clawed toad (Xenopus), with a ZZ/ZW system, a paralogue of DMRT1 on the W chromosome (DM-W) acts as an ovary-determining factor [Yoshimoto et al., 2008; Yoshimoto et al., 2010]. In female gonads, DM-W opposes DMRT1 and induces expression of FOXL2 and aromatase [Piprek et al., 2018]. Over-expression of DM-W in ZZ tadpoles upregulates expression of FOXL2 and aromatase, while over-expression of DMRT1 in ZW tadpoles leads to masculinization of the gonads. Steroid treatment has also been shown to induce gonadal sex-reversal in a number of amphibian species [Nakamura, 2010; Piprek et al., 2012] [Miura et al., 2016; Flament, 2016]. In several reptile species with temperature-sensitive sex determination, treatment with exogenous estrogen results in all female offspring, while treatment with aromatase inhibitors results in all male offspring [Pieau and Dorizzi, 2004; Barske and Capel, 2010; Kohno et al., 2015]. Although estrogen has been shown to have a key role in gonadal development in a wide variety of non-mammalian vertebrates, estrogen does not affect gonadal development in mammals, with the possible exception of goats and marsupials: in goats, aromatase is a downstream target of FOXL2, and loss of FOXL2 induces female-to-male sex-reversal and decreased aromatase levels [Pailhoux et al., 2001, 2002; Pannetier et al., 2006; Boulanger et al., 2014], while gonadal development in marsupials occurs around the time of birth, and exogenous estrogen treatment of the young in the pouch has been shown to cause male-to-female gonadal sex-reversal [Coveney et al., 2001; Renfree and Shaw, 2001; Pask et al., 2010].

## DMRT1

**Fig. 4.**
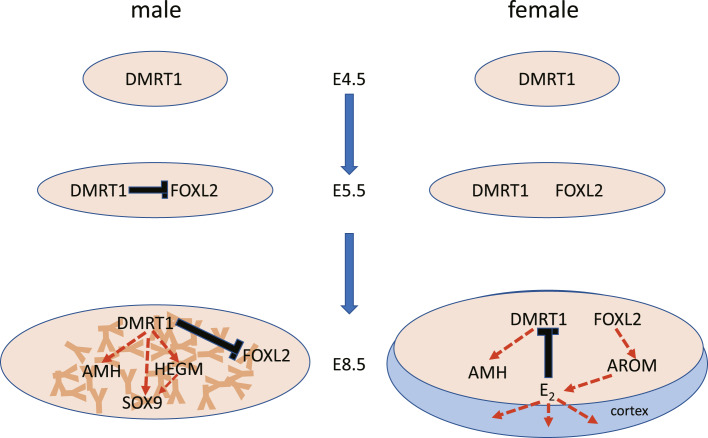
Schematic illustrating expression patterns and possible molecular interactions in the developing testis and ovary AROM, aromatase; E_2_, estradiol).

Although DMRT1 is clearly essential for testis development, the molecular mechanism underlying this developmental process is not well understood. For example, it is theoretically possible that different DMRT1 transcripts are expressed in male and female gonads as the DMRT1 locus has been shown to generate a number of different alternatively spliced transcripts [Zhao et al., 2007; Zhao et al., 2022]. This now seems unlikely as a recent study has shown that only a single DMRT1 transcript is expressed in the developing chick gonads, and that the same transcript is expressed in male and female gonads [Zhao et al., 2022]. In any event, it is widely assumed that testis-promoting effects of DMRT1 dosage are due to elevated levels of DMRT1 protein in male gonads compared to female gonads at the point of sex determination (between E4.5 and E6.5). This assumption is based on a number of studies that have shown that DMRT1 transcript levels are at least two-fold higher in male gonads than in female gonads throughout the period E4.5–E9.5 [Raymond et al., 1999; Smith et al., 2003; Omotehara et al., 2014] (Fig. 2). However, it may not be as simple as “more DMRT1 RNA = more DMRT1 protein” [Zhao et al., 2022]. This study found that, although DMRT1 transcript levels were higher in males than in females, DMRT1 protein levels were equivalent in male and female gonads during the early stages of gonadal development. This study also showed that, from E6.5 onward, DMRT1 protein levels were indeed higher in male gonads than in female gonads, but importantly, suggested that this sex difference was due to a reduction in DMRT1 transcription and protein levels in female gonads, rather than the expected increase in DMRT1 protein levels in male gonads. The reduction in DMRT1 protein levels in the developing female gonads coincided with the onset of estrogen synthesis [Guioli et al., 2014], and the authors examined the effects on DMRT1 protein levels of suppressing estrogen synthesis in embryonic female gonads. The aromatase inhibitor fadrozole was used to suppress aromatase activity and generate a series of female embryos with ovo-testes. In these female (ZW) gonads, areas that lacked aromatase expression showed elevated levels of DMRT1 protein and sex cord formation. It is well documented that in the early stages of gonadal development DMRT1 expression is induced in both male and female gonads [Raymond et al., 1999; Smith et al., 2003; Omotehara et al., 2014; Hirst et al., 2018] and perhaps the role of DMRT1 at this stage is to initiate medullary differentiation in both sexes. Later, in females, FOXL2 is thought to induce the expression of aromatase which catalyzes the conversion of androgens to estrogens, promoting the development of the ovarian cortex. Maybe estrogens also suppress expression of DMRT1 and prevent differentiation of the medullary sex cords. In male gonads, FOXL2 expression is not induced and DMRT1 levels are maintained promoting the differentiation of sex cords and Sertoli cells and the production of AMH and SOX9. Under this scenario, the testis pathway could be considered the “default” option, while ovary development would require induction of FOXL2 expression and estrogen synthesis (Fig. 4). The key remaining question is, how does expression of DMRT1 (directly or indirectly) block expression of FOXL2 if not through elevated levels of DMRT1 protein?

## Other Factors Involved in Gonad Differentiation

### FOXL2

**Fig. 5.**
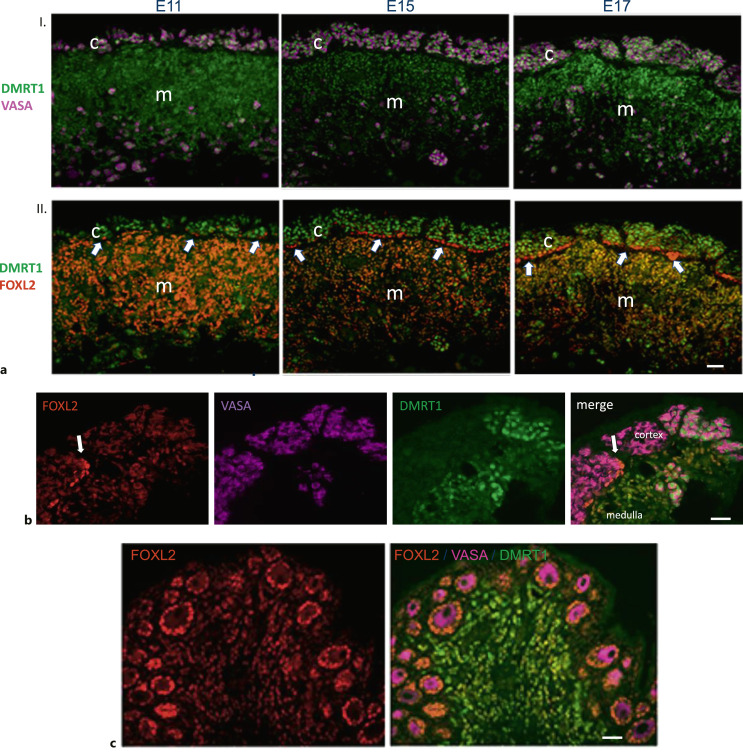
Expression of FOXL2 in subcortical region of the developing ovary. **a** IHC showing expression of (I.) DMRT1 (green) and VASA (magenta), and (II.) DMRT1 and FOXL2 (red) in histological sections of the developing ovary at E11, E15, and E17. FOXL2 expression in the medulla diminishes with development except in the subcortical region. Arrows indicate cells expressing elevated levels of FOXL. From a small number of isolated cells at E11, the population of cells expressing high levels of FOXL2 expands into a near-continuous layer between the cortex and medulla at E17. **b** IHC showing expression of FOXL2 (red), VASA (magenta), and DMRT1 (green) in a histological section of the developing ovary at E20. Arrow indicates highly expressing FOXL2 cells infiltrating cortex. **c** IHC showing expression of FOXL2 (red), VASA (magenta), and DMRT1 (green) in histological section through epithelial fold on ovarian surface 2 weeks post-hatch. Germ cells/ova are surrounded by a single layer of FOXL2-expressing cells.

FOXL2, a member of the forkhead box family of transcription factors, has been shown to be a female sex-determining gene in the goat [Pailhoux et al., 2001; Boulanger et al., 2014]. In the mouse embryonic gonads, FOXL2 expression is female-specific and can be detected shortly after the point of sex determination [Wilhelm et al., 2009]. Later in development, FOXL2 expression is found in theca and granulosa cells and their precursors. In the chicken, FOXL2 is only detected in female embryos and expression is induced from the early stages of gonadal development (around E6) [Loffler et al., 2003; Govoroun et al., 2004; Hudson et al., 2005; Major et al., 2019]. FOXL2 expression is found in cells of the female medulla, immediately prior to, and co-localized with aromatase (CYP191A) expression. FOXL2 has been shown to be a direct activator of aromatase in other species [Pannetier et al., 2006; Wang et al., 2007; Fleming et al., 2010], so it is possible that, in chickens, the activation of FOXL2 leads to the synthesis of estrogens and differentiation of the ovarian cortex. It is worth noting that, in the chicken, other factors may be required [Major et al., 2019]. However, although all aromatase +ve cells express FOXL2, not all FOXL2 cells express aromatase. Around E11, as previously described [Major et al., 2019], a small number of isolated cells in the region between the cortex and the medulla begin to express high levels of FOXL2 (Fig. 5). Over the next 10 days, the numbers of cells expressing high levels of FOXL2 increase until this population forms a near-continuous layer in the subcortical region. In the days immediately prior to hatch, this population of cells appears to infiltrate the upper cortex and enclose nests of germ cells. In the post-hatch period, individual germ cells/ova are surrounded by a layer of cells expressing high levels of FOXL2 – presumably granulosa cells.

### R-Spondin 1

R-spondin 1 (RSPO1) together with WNT4 is a component of the WNT signaling pathway and induces activation of β-catenin. RSPO1 mutations have shown that this factor has an important role in mammalian ovarian differentiation, but the exact mechanism is unresolved [Chassot et al., 2014]. In the chicken embryo, RSPO1 is expressed throughout the ovary until around E7 when expression is restricted to the outer cortical layer [Ayers et al., 2013b]. Fadrozole-induced sex-reversal experiments suggest that RSPO1 expression is influenced by estrogen levels [Smith et al., 2008]. It seems likely that RSPO1, WNT4, and β-catenin play a role in regulating proliferation of the ovarian cortex.

### Hemogen

The Z chromosome gene hemogen (HEGM) is implicated in testis differentiation in the chicken. HEGM is so named due to its association with hematopoiesis in mammals. HEGM is expressed at high levels in the developing testes from around E5.5 until E8.5, and then, levels decrease significantly [Nakata et al., 2013]. Over-expression of HEGM in females leads to the upregulation of DMRT1 and downregulation of FOXL2, and fadrozole-induced ovary-to-testis sex-reversal leads to the upregulation of HEGM expression. HEGM expression initiates slightly later than that of DMRT1, but well before expression of SOX9 is detected. It is worth noting that male-specific expression of HEGM has not been observed in other avian species [Hirst et al., 2017].

## Cell Autonomous Sex Identity (CASI)

Until recently, overall sexual development in vertebrates was assumed to follow principles based on the mammalian model, where the sexual phenotype of an individual depends on the gonad: i.e., the somatic cells of male and female tissues are sexually indifferent, and tissue sexual dimorphisms are imposed by gonadal hormones. Aspects of songbird neural development and marsupial development were identified as specific examples that did not conform to these principles [Renfree and Short, 1988; O et al., 1988; Arnold, 1997; Wade and Arnold, 2004], but these were considered exceptions. However, a series of studies have now established that these principles do not apply to chickens, and that somatic chicken cells possess an inherent sex identity. Convincing evidence came from the study of gynandromorph birds [Agate et al., 2003; Zhao et al., 2010; Morris et al., 2018]. These birds display a marked bilateral symmetry: one side of the bird appears phenotypically male, and the other side appears phenotypically female. Surprisingly, examination of cells from tissues on both sides of these animals revealed that these animals were genuine male:female chimeras [Zhao et al., 2010]. It transpired that tissues that contained mostly male cells were of male appearance and tissues with a preponderance of female cells appeared female. This suggested that male (ZZ) tissues and female (ZW) tissues were either not responding to gonadal hormones or responding in different ways to the same hormones. To explore this further, a series of embryos with mixed-sex chimeric gonads was generated. Examination of the medullary somatic cells in the chimeric gonads revealed that donor tissue retained donor identity within the host gonad. Female cells transplanted into a male host formed a female medulla with FOXL2 and aromatase expression within the male gonad. Conversely, male cells transplanted into a female host formed a male medulla containing sex cords with Sertoli cells that expressed AMH and SOX9. It is clear from these studies that the concept that gonadal hormones impose sexual dimorphisms does not apply to chickens and that chicken somatic cells have a CASI. The principle of CASI was reinforced by the recent CRISPR-cas9 study involving gene editing of DMRT1 in chickens [Ioannidis et al., 2021]. In this study, male chickens with only a single functional copy of DMRT1 developed ovaries instead of testes. These birds were hatched and raised to sexual maturity. With the exception of primary reproductive tissues, the sexual phenotype of these male birds with ovaries was identical to that of wild-type male birds. The growth rate and weight at sexual maturity of these birds were also identical to that of wild-type males. It is clear from these analyses that sexual dimorphisms such as external appearance and muscle mass result from the CASI of avian somatic cells, and not from the influence of gonadal hormones.

**Fig. 6.**
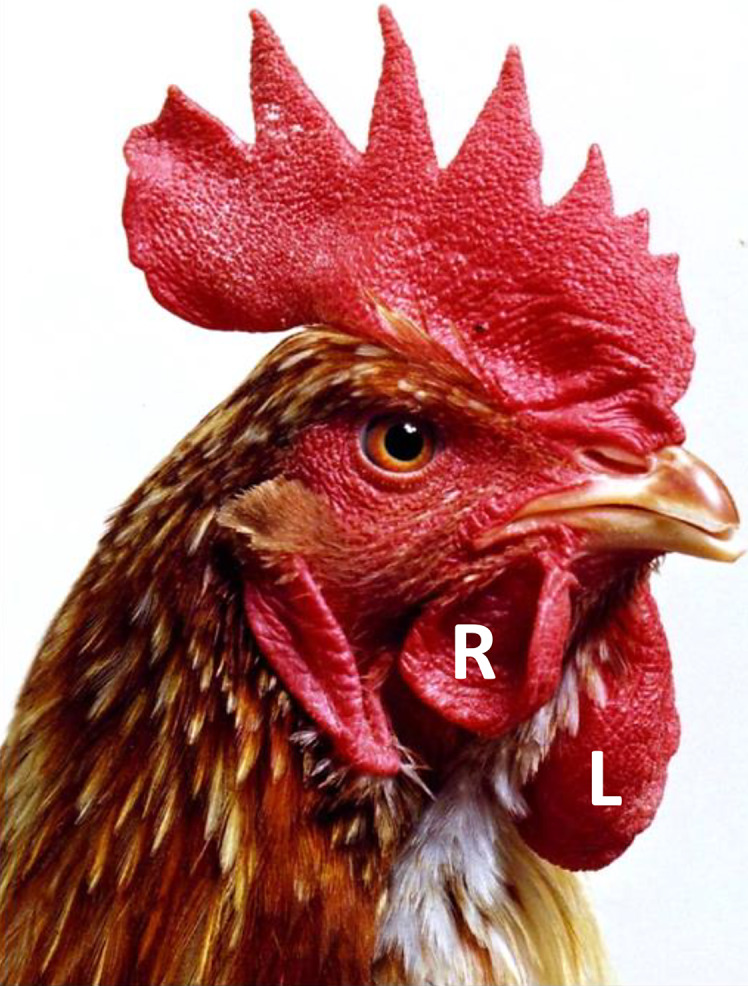
Photograph of adult gynandromorph chicken. Bird showed bilateral asymmetry with left side composed primarily of male (ZW) cells and right side composed primarily of female (ZW) cells. The image clearly shows that the wattle on the male (left) side is considerably larger than the wattle on the female (right) side. Post-mortem weight of left wattle was four times greater than that of right wattle. R, right; L, left.

Although steroid hormones play a significant role in gonadal development in the chicken, they do not contribute significantly to secondary sexual dimorphisms which seem to result directly from male and female CASI. This conclusion may appear at odds with many historical reports that have described tissues such as the wattles and spurs as “hormone-sensitive” tissues. However, given recent findings, it seems likely that the reported effects were due to non-physiological levels of exogenous hormones [Zhao et al., 2010; Ioannidis et al., 2021]. The fact that the wattles of male birds with only a single functional copy of DMRT1 (and with ovaries) are identical to those of wild-type male birds demonstrates that the switch from physiological levels of testicular hormones to physiological levels of ovarian hormones does not influence wattle growth [Ioannidis et al., 2021]. This is also supported by the dramatically different sized wattles on the right and left sides of gynandromorph birds, despite the fact that both sides are exposed to the same hormonal milieu [Zhao et al., 2010] (Fig. 6). As chicken cells possess CASI, efforts to generate larger female birds by gonad sex-reversal (a recurring theme in poultry production) are likely to be unsuccessful.

## Avian Ovarian Follicle

**Fig. 7.**
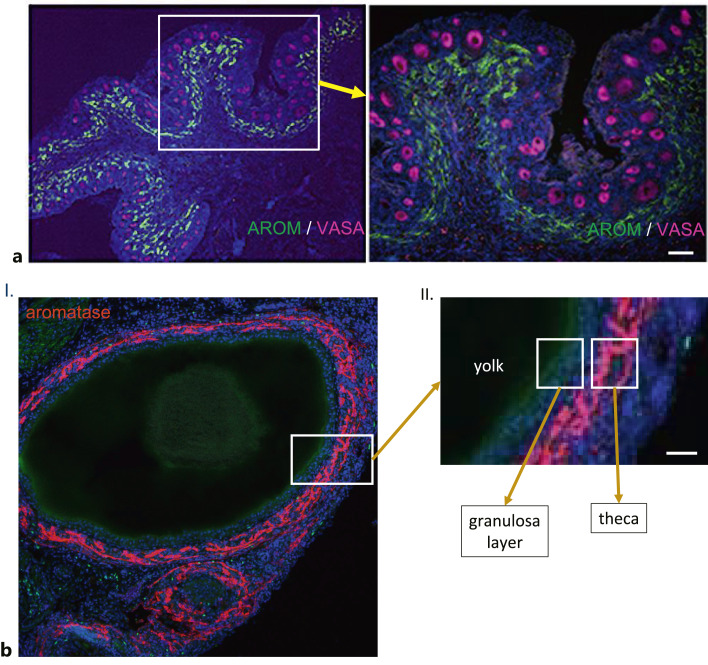
Aromatase expression in the post-hatch ovary. **a** IHC of aromatase (green) and VASA (magenta) expression in histological section through epithelial folds on surface of 2 weeks post-hatch ovary. Aromatase is highly expressed in the subcortical region, in the medullary cells lining the epithelial folds. Aromatase-expressing cells appear flattened and elongated. **b** IHC showing aromatase (red) expression in small follicles of sexually mature female chicken (I.). Magnified region of follicular wall (II.). Ovum is surrounded by a granulosa layer only a few cells in thickness. Aromatase expression is largely restricted to theca cells and absent from granulosa cells.

Although the terminology used to describe ovarian structures and cell types is conserved across different vertebrates, the physical dynamics of ovarian function are dramatically different [Sturkie’s Avian Physiology, 6th edition, Scanes and Dridi, 2014]. For example, during each estrous cycle in mammals, waves of primary follicles initiate follicular growth and then undergo atresia. During one such wave, a small number of follicles are selected to complete folliculogenesis and ovulation occurs once per estrous cycle. In contrast, ovulation occurs daily in commercial chickens. The functionally mature chicken ovary contains a large pool of small (1–5 mm diameter) white cortical follicles, a smaller number of slightly larger (5–10 mm) yellow follicles, and 5–6 large (10–40 mm) yellow preovulatory follicles. The yellow follicles are arranged in a hierarchical order that clearly indicates the stage of the development of each follicle, with the largest follicle the most mature and the next in line to ovulate. Following ovulation, a single follicle is selected from the pool of small yellow follicles each day to join the preovulatory follicle hierarchy, and follicular recruitment from the cohort of white follicles into the small yellow follicle group is also a continuous process. The structure of the mature follicle also differs dramatically between mammals and birds. In mammals, there are multiple layers of granulosa cells surrounding a fluid-filled cavity containing the cumulus oophorus and the ovum. In the avian mature follicle, there is no antrum and no cumulus – the yolk-filled ovum fills the entire follicle. During development, the diameter of the oocyte/ovum increases in size from microns to centimeters. The preovulatory ovum is surrounded by a few layers of cuboidal granulosa cells, a basement membrane, and layers of flattened theca cells. While chicken granulosa cells fit the broad description of “cells that surround and nurture the maturing oocyte,” they do not conform to definitions that include “cells that produce estrogen and become luteal cells after ovulation.” Aromatase-expressing cells in the embryonic gonadal medulla have been considered progenitors of the granulosa cells, but this is not the case. Post-hatch, these aromatase-expressing cells form the core of the cortical folds on the surface of the ovary and it seems likely that these cells are destined to become the theca layers of the maturing follicles (Fig. 7a). In the chicken ovary, although the granulosa cells express FOXL2 [Govoroun et al., 2004], they do not express aromatase and estrogen synthesis occurs in the cells of the thecal layer (Fig. 7b).

## Bipotential or Binary?

**Fig. 8.**
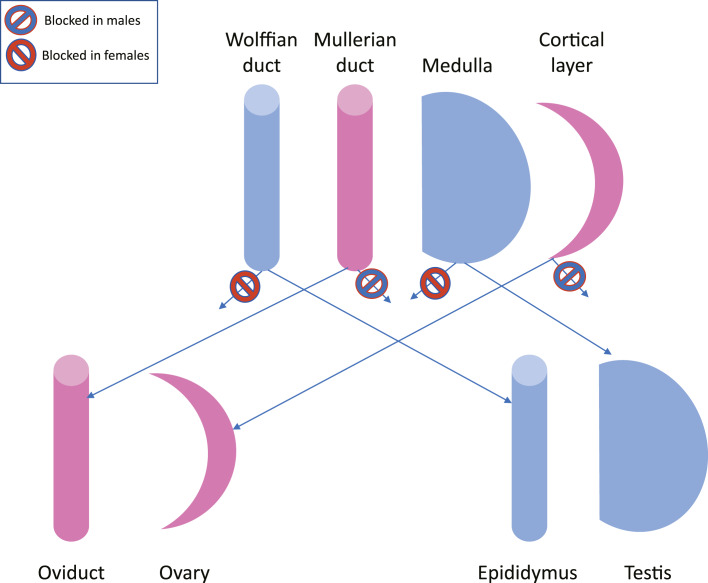
Hypothetical alternative to the concept of bipotential gonad.

The mouse genital ridge is frequently described as “bipotential,” i.e., a single undifferentiated tissue capable of differentiating into either of two distinct organs. Expression of the Sry gene at the appropriate stage of development determines a testicular rather than an ovarian fate. Again, the mammalian model is thought to apply across vertebrates, but in the chicken at least, this model does not seem to be a perfect fit. In the initial stages of gonadogenesis, it may be that the left gonads of both male and female embryos initiate differentiation of both a testis (primitive sex cords in medulla) and an ovary (thickened epithelium on the left gonad). At this point, one of the tissues is selected for full development while the alternative tissue remains undeveloped. In the case of the chicken, this selection may be based on estrogens: if estrogens are present, cortex development is promoted and medullary cord formation is inhibited; without estrogens, sex cords develop and the epithelium thins. Perhaps gonadal development in the chicken can be considered in the same light as the progenitors of the reproductive tract – the Wolffian and Mullerian ducts: both are present, and one is selected in each sex? Specific hormones determine whether the Wolffian duct or the Mullerian duct is retained, to develop into either the epididymis/vas deferens in males or the oviduct in females (Fig. 8). Under this scenario, it may be more appropriate to describe the gonadal precursor in the chicken as a “binary” tissue rather than as “bipotential.”

## Acknowledgments

We thank the National Avian Research Facility at the Roslin Institute for animal husbandry services and the provision of eggs, and the Bioimaging facility for technical assistance. We thank M. A. Hattori for kindly providing VASA antibody; S. Guioli, R. Lovell-Badge, and C. Smith for DMRT1 antibodies; and N. Russell for photography.

## Conflict of Interest Statement

The authors declare that there are no conflicting interests.

## Funding Sources

Funding for this work was from the Biotechnology and Biotechnology Research Council (BB/N018672/1 and BB/E015425/1) and the Roslin Institute Integrated Strategic Programme.

## Author Contributions

Michael Clinton prepared the manuscript and designed the figures. Debiao Zhao compiled the figures and edited the manuscript.
