# Supplementary material for: Avian Sex Determination: A Chicken and Egg Conundrum
Source: Sex Dev. 2023 Feb 16;17(2-3):120–33. doi: 10.1159/000529754 (PMC10659007; doi:10.1159/000529754)
Supplement: Supplementary file 1 [file Supplementary_material-Suppl.1-s1.pptx]

## Slide 1
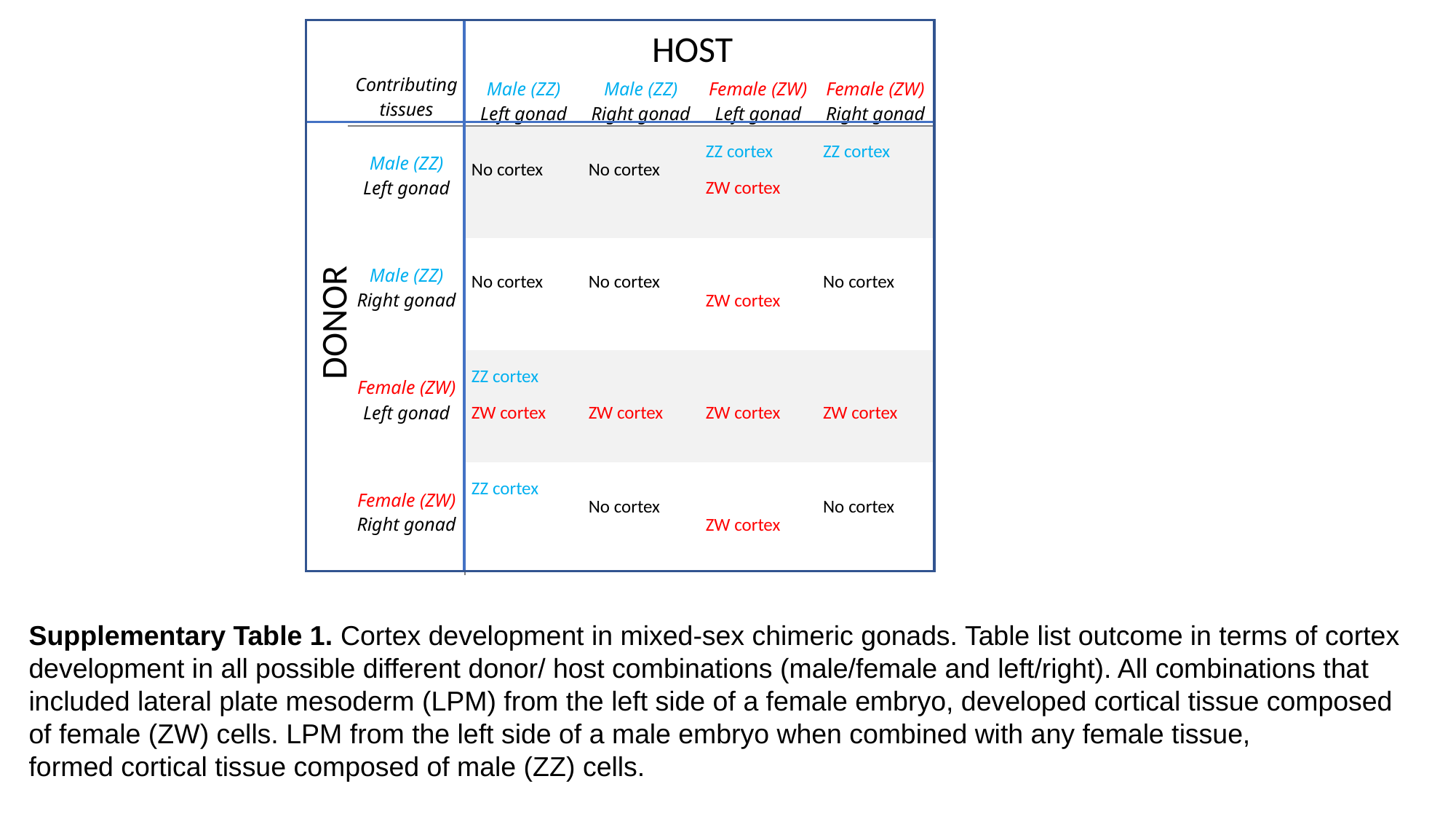

HOST
| Contributing tissues | Male (ZZ) Left gonad | Male (ZZ) Right gonad | Female (ZW) Left gonad | Female (ZW) Right gonad |
| --- | --- | --- | --- | --- |
| Male (ZZ) Left gonad | No cortex | No cortex | ZZ cortex   ZW cortex | ZZ cortex |
| Male (ZZ) Right gonad | No cortex | No cortex | ZW cortex | No cortex |
| Female (ZW) Left gonad | ZZ cortex   ZW cortex | ZW cortex | ZW cortex | ZW cortex |
| Female (ZW) Right gonad | ZZ cortex | No cortex | ZW cortex | No cortex |
DONOR
Supplementary Table 1. Cortex development in mixed-sex chimeric gonads. Table list outcome in terms of cortex
development in all possible different donor/ host combinations (male/female and left/right). All combinations that
included lateral plate mesoderm (LPM) from the left side of a female embryo, developed cortical tissue composed
of female (ZW) cells. LPM from the left side of a male embryo when combined with any female tissue,
formed cortical tissue composed of male (ZZ) cells.
